# Seasonal and Spatial Variations in Bacterial Communities From Tetrodotoxin-Bearing and Non-tetrodotoxin-Bearing Clams

**DOI:** 10.3389/fmicb.2020.01860

**Published:** 2020-08-05

**Authors:** Laura Biessy, John K. Pearman, Kirsty F. Smith, Ian Hawes, Susanna A. Wood

**Affiliations:** ^1^Coastal and Freshwater, Cawthron Institute, Nelson, New Zealand; ^2^Department of Biological Sciences, University of Waikato, Hamilton, New Zealand; ^3^New Zealand Food Safety Science and Research Centre, Palmerston North, New Zealand

**Keywords:** bacteria, biotoxin, bivalves, cyanobacteria, metabarcoding, microbiome, high-throughput sequencing, *Paphies australis*

## Abstract

Tetrodotoxin (TTX) is one of the most potent naturally occurring compounds and is responsible for many human intoxications worldwide. *Paphies australis* are endemic clams to New Zealand which contain varying concentrations of TTX. Research suggests that *P. australis* accumulate the toxin exogenously, but the source remains uncertain. The aim of this study was to identify potential bacterial TTX-producers by exploring differences in bacterial communities in two organs of *P. australis*: the siphon and digestive gland. Samples from the digestive glands of a non-toxic bivalve *Austrovenus stutchburyi* that lives amongst toxic *P. australis* populations were also analyzed. Bacterial communities were characterized using 16S ribosomal RNA gene metabarcoding in *P. australis* sourced monthly from the Hokianga Harbor, a site known to have TTX-bearing clams, for 1 year, from ten sites with varying TTX concentrations around New Zealand, and in *A. stutchburyi* from the Hokianga Harbor. Tetrodotoxin was detected in *P. australis* from sites all around New Zealand and in all *P. australis* collected monthly from the Hokianga Harbor. The toxin averaged 150 μg kg^–1^ over the year of sampling in the Hokianga Harbor but no TTX was detected in the *A. stutchburyi* samples from the same site. Bacterial species diversity differed amongst sites (*p* < 0.001, *F* = 5.9) and the diversity in siphon samples was significantly higher than in digestive glands (*p* < 0.001, *F* = 65.8). Spirochaetaceae (4–60%) and Mycoplasmataceae (16–78%) were the most abundant families in the siphons and the digestive glands, respectively. The bacterial communities were compared between sites with the lowest TTX concentrations and the Hokianga Harbor (site with the highest TTX concentrations), and the core bacterial communities from TTX-bearing individuals were analyzed. The results from both spatial and temporal studies corroborate with previous hypotheses that *Vibrio* and *Bacillus* could be responsible for the source of TTX in bivalves. The results from this study also indicate that marine cyanobacteria, in particular picocyanobacteria (e.g., *Cyanobium*, *Synechococcus*, *Pleurocapsa*, and *Prochlorococcu*s), should be investigated further as potential TTX producers.

## Introduction

Tetrodotoxin (TTX) is one of the most potent naturally occurring neurotoxins in the world (Noguchi et al., 2011). This toxin was thought to occur only in tropical to sub-tropical climates but has now been reported from more temperate climates in the Pacific and Mediterranean (Lago et al., 2015; Biessy et al., 2019a). Tetrodotoxin is found in a wide variety of phylogenetically unrelated terrestrial, marine and freshwater organisms (Bane et al., 2014) and the consumption of seafood, primarily puffer fish and gastropods, contaminated with TTX has resulted in many human intoxications (Noguchi et al., 2011). It was first identified in marine bivalves (Japanese scallops, *Pactinopecten yessoensis*) in 1993 (Kodama et al., 1993), but recently there has been an increase in detection and published reports with TTX now identified in ten bivalve species from seven countries (Biessy et al., 2019a). The detection of TTX in harvested edible bivalves has led to concerns about health risks for human consumers and prompted further studies worldwide.

Despite substantial research over three decades, the origin of TTX remains uncertain (Chau et al., 2011). The two most common hypotheses regarding its origin are: (1) it is endogenously produced by symbiotic bacteria, or (2) it is exogenously accumulated through dietary sources (e.g., bacteria, micro-algae). Exogenous or symbiotic bacteria are commonly touted as the most likely TTX-producers, with a wide diversity of species and strains implicated. There are reports in the literature of at least 150 TTX-producing bacterial strains, with *Vibrio* species comprising more than 30% of records, followed by *Bacillus*, *Pseudomonas*, *Actinomyces*, and *Micrococcus* (Turner et al., 2018; Katikou, 2019). However, it is estimated that less than 1% of all bacteria are culturable, making isolation, culturing and confirmation of toxin production in bacteria a significant challenge (Chau et al., 2011). Studies have claimed to isolate bacteria that potentially produce TTX, but the concentration of toxin identified in laboratory cultures is usually low compared to amounts in the host animals. For example, 184 ng g^–1^ of TTX was reported from an isolated *Vibrio* sp. in comparison to 36 μg g^–1^ in the tissue of its host, the sea snail *Nassarius semiplicatus* (Wang et al., 2008). Additionally, there are indications that some of the methods (e.g., high performance-liquid chromatography and gas chromatography-mass spectrometry) used to analyze the TTX-producing bacteria in culture generate false positives (Matsumura, 1995).

Limited research has been undertaken on the source of TTX in marine bivalves but, as in other organisms, available evidence indicates an exogenous source (Biessy et al., 2018, 2019a). Gammaproteobacteria, particularly *Vibrio* and *Pseudomonas* species, have been linked to the accumulation of TTX in bivalves, with two recent studies finding correlations between the presence of *Vibrio*, *Pseudomonas* and TTX in shellfish (Turner et al., 2017b; Leão et al., 2018). However, these studies were unable to culture any TTX-producing bacteria from the shellfish samples. The hypothesis that bacteria or micro-algae are the source of TTX in these organisms is also fueled by reports of toxic episodes in bivalves being more prevalent during warmer months, particularly in late spring in Europe (Gerssen et al., 2018; Leão et al., 2018) and New Zealand (Biessy et al., 2019b). This may indicate seasonal changes in bacterial communities and an increase in warm-water adapted TTX-producing microorganisms.

The application of molecular genetics-based techniques to characterize entire communities (e.g., metabarcoding) is escalating rapidly (Taberlet et al., 2012; Deiner et al., 2017). Metabarcoding is a culture-independent technique that enables taxa in an environmental sample or organ to be identified simultaneously and has previously been used as a tool to investigate the source of TTX by characterizing the core bacterial communities of toxin-bearing organisms. Salvitti et al. (2017) used metabarcoding to investigate the foregut content of the highly toxic, carnivorous sea-slug *Pleurobranchaea maculata* from New Zealand. The authors found a high abundance of sequences taxonomically related to Cnidaria and Annelida, taxa known to contain neurotoxins that could have been ingested by *P. maculata*. Turner et al. (2018) studied the bacterial communities of the TTX-bearing nemertean worm *Cephalothrix simula* and showed the prevalence of a large number of bacterial genera previously associated with TTX production including *Alteromonas*, *Vibrio* and *Pseudomonas*. In a recent study, Melnikova and Magarlamov (2020) investigated the core bacterial communities of TTX-bearing and non-bearing nemertean worms from Japan and demonstrated that toxic individuals tend to have more potential TTX-producing bacterial strains (e.g., *Pseudomonas*, *Vibrio* and *Bacillus*) in their microflora but they were unable to identify a possible TTX producer. One issue with microbiome analyses of motile organisms is that it only provides a “snapshot” of what the organisms have ingested at one point in time and location. These organisms may move from the site of TTX-ingestion, making it challenging to identify TTX sources in the environment. Analysis of bacterial communities of sedentary animals, such as marine bivalves, would provide novel insights into the source of TTX, especially if investigated over spatial and temporal scales.

In 2011, McNabb et al. (2014) recorded high concentrations (800 μg kg^–1^) of TTX in an endemic marine clam *Paphies australis* in Whangapoua (Coromandel, New Zealand). Immunohistochemistry and chemical analyses of *P. australis* individuals from Hokianga Harbor (Northland, New Zealand) revealed that the siphons and organs involved in feeding contained the highest concentrations of TTX (means of 403 and 34 μg kg^–1^, respectively), providing evidence to support the hypothesis of an exogenous source (Biessy et al., 2018). This hypothesis was further strengthened during an experiment where *P. australis* individuals were maintained in captivity for 150 days and showed significant depuration of TTX. Tetrodotoxin present in the digestive glands at the start of the experiment depurated rapidly and only traces remained after 21 days (Biessy et al., 2019b). Furthermore, *P. australis* sourced from different locations around New Zealand have significant variations in TTX concentrations (Biessy et al., 2019b). The bivalves collected from warmer sites (at lower latitudes) contained higher TTX concentrations than the ones from the colder regions.

The overarching goal of the present study was to further investigate the source of TTX in *P. australis.* We used metabarcoding to characterize bacterial communities in the digestive glands and siphons sourced monthly from one site with high concentrations, for 1 year, and from single time points at ten sites with *P. australis* that contained varying TTX concentrations. The aims of the study were: (1) to explore differences in bacterial communities between *P. australis* populations with high and low TTX concentrations and a non-toxic bivalve species, the cockle *Austrovenus stutchburyi*, found in the same habitat at one site; (2) to evaluate the core bacterial communities of *P. australis* at one site where TTX concentrations were relatively stable over time; and (3) use the genetic information from bacterial amplicon sequence variants and corresponding toxin data to identify potential TTX-producing bacterial species. Intra- and inter-species variation in toxin concentration provides a unique opportunity to examine the differences in bacterial communities of these populations and identify any specific bacterial communities elements correlated with toxin concentrations in the host.

## Materials and Methods

### Sample Collection

#### Spatial Study

*Paphies australis* (*n* = 8 per site) were collected from ten sites spanning the length of New Zealand between September 2017 and February 2019 (Supplementary Figure S1). The *P. australis* were chilled (ca. 8°C) and sent overnight to the laboratory (Cawthron Institute, Nelson, New Zealand). Once in the laboratory, three of the eight individuals were aseptically dissected for genetic analysis: the siphons and digestive glands were placed in individual tubes and stored frozen (−20°C) until further analysis. The remaining five *P. australis* from each site were used for toxin extraction. *Austrovenus stutchburyi* were identified amongst the *P. australis* beds in the Hokianga Harbor and were also collected (*n* = 8) in September 2017 for further analysis. Five *A*. *stutchburyi* were used whole for toxin extraction and the remaining (*n* = 3) were aseptically dissected and the digestive glands were placed in individual tubes and stored frozen (−20°C) until DNA was extracted.

#### Temporal Study

*Paphies australis* were collected from the Hokianga Harbor (Northland, New Zealand, 35°28′S, 173°24′E; Supplementary Figure S1) monthly from April 2017 to March 2018 (except May 2017, *n* = 11 sampling events), chilled and sent to the laboratory overnight. *P. australis* from this location had previously been shown to contain TTX (Biessy et al., 2018). *P. australis* used for the temporal study were collected as part of a larger survey investigating TTX concentrations in recreationally harvested bivalves in New Zealand (Harwood et al. in preparation). The *P. australis* received each month were pooled together to follow the standard operating procedure which is used in the accredited laboratory that undertook the analysis of these samples. Three *P. australis* were put aside each month and were then dissected and stored for further genetic analysis as described above.

### Toxin Extractions

Tetrodotoxin extraction was performed as previously described in Biessy et al. (2018). Briefly, for the spatial samples, *P. australis* and *A. stutchburyi* were shucked and individually weighed (ca. 0.5–3.0 g) and placed in a sterile tube (50 mL) with a corresponding volume (ca. 500–3,000 μL) of Milli-Q water containing 1% acetic acid. Organisms were homogenized (Ultra-Turrax^®^, IKA^®^, NC, United States) for 45 s. The temporal samples were freshly opened, left to drain (5 min), pooled together for homogenisation (Ultra-Turrax^®^) for 5 min and a sub-sample was weighed (5 g) and placed in a sterile tube containing Milli-Q water and 1% acetic acid (5 mL). Subsequently extraction for both the spatial and temporal samples followed the same procedure. The tubes were boiled (5 min) and cooled in an ice bath (5 min) before briefly vortexing. Samples were centrifuged (3,200 × *g*, 10 min) and 0.5–1 mL of the supernatant transferred to a centrifuge tube (1.7 mL) containing 25% ammonia (2.5–5 μL; Honeywell, Seelze, Germany). Samples were then centrifuged (17,000 × *g*, 1 min) and the supernatant subjected to the graphitised carbon solid phase extraction (SPE) method as described in Boundy et al. (2015) using Supelclean ENVI-Carb 250 mg/3 mL cartridges (Sigma-Aldrich, MO, United States). Tetrodotoxin was analyzed and quantified by liquid chromatography tandem-mass spectrometry analysis as described by Turner et al. (2017a). The toxin results for samples collected in 2017 and 2018 were previously published by Biessy et al. (2019b) but are integrated with the molecular dataset in this study.

#### Statistical Analysis

Statistical analyses for toxin concentrations were performed using the R statistical package (R-Project, 2019). Normality was checked through inspection of Quantile-Quantile plots and conducting a Shapiro-Wilk test. As the data deviated from a normal distribution the spatial data was log transformed. Levene’s test was used to assess the equality of variance in TTX concentrations between sites (Carroll and Schneider, 1985). Statistical differences in TTX concentrations between sites was assessed using a one-way analysis of variance (ANOVA; Factor: Site; 10 levels). A Tukey’s honestly significant difference (HSD) *post-hoc* test was used to identify which sites were responsible for the significant differences.

### Metabarcoding

#### DNA Extractions and PCR

Dissected organs from both studies were individually placed in the first tube containing bashing beads of the DNA extraction kit (DNeasy Powersoil Pro kit, Qiagen, MD, United States). The DNA was then extracted following manufacturer’s instructions using an automated homogenizer (1600 MiniG Automated Tissue Homogenizer and Cell Lyser, SPEX SamplePrep, NJ, United States) and a robotic workstation for DNA extraction (QIAcube, Qiagen). Negative extraction controls were performed every 23 samples. Amplification and sequencing PCR Amplicons were generated covering the V3 and V4 regions of the 16S ribosomal RNA (rRNA) gene using the primer sets as described in Klindworth et al. (2013) and were modified to include Illumina^TM^ overhang adaptors following the dual-indexing method from Kozich et al. (2013): Bact341F-5′–TCG TCG GCA GCG TCA GAT GTG TAT AAG AGA CAG CCT ACG GGN GGC WGC AG–3’ and Bact785R-5′–GTC TCG TGG GCT CGG AGA TGT GTA TAA GAG ACA GGA CTA CHV GGG TAT CTA ATC C–3′. PCR reactions were undertaken in triplicate with 450 nM of each primer, 25 μL of 2X MyFi^TM^ Mix (Bioline, United Kingdom), ca. 5 ng of DNA, and sterile water for a total reaction volume of 50 μL. Cycling conditions were: 95°C for 5 min, followed by 30 cycles of 94°C for 30 s, 54°C for 30 s, 72°C for 45 s, and a final extension of 72°C for 7 min. Triplicates of PCR products were pooled and visualized on 1.5% agarose gel with Red Safe^TM^ DNA Loading Dye (Herogen Biotech, United States) and UV illumination. PCR negatives were run to assess for contamination during the PCR steps. The PCR products were purified, cleaned of primer dimers and normalized using SequalPrep Normalization plate (Thermo Fisher, MA, United States), and submitted to Auckland Genomics (University of Auckland, New Zealand) for library preparation. Sequencing adapters and sample-specific indices were added to each amplicon via a second round of PCR using the Nextera^TM^ Index kit (Illumina Inc., United States). Amplicons were pooled into a single library and paired-end sequences (2 × 250 bp) generated on a MiSeq^®^ instrument. The sequencing libraries were prepared following the Illumina 16S Metagenomics Library Prep manual with the exception that after the indexing, 5 μL of each sample (including three water samples acting as sequencing blank) was pooled and a single clean-up was undertaken on the pool instead of samples being individually cleaned. Quality control was undertaken using a bioanalyzer before the library was diluted to 4 nM and denatured. A 15% PhiX spike was used and the final loading concentration was 7 ρM. Sequence data were automatically demultiplexed using MiSeq^®^ Reporter (version 2, Illumina Inc.), and forward and reverse reads assigned to samples. Raw sequence reads were deposited in the National Center for Biotechnology Information (NCBI) short read archive under the accession number PRJNA623388.

#### Bioinformatics

##### Amplicon sequence variant inference and taxonomic assignments

Raw reads were processed, subsequent to primers being removed with *cutadapt* (Martin, 2011), using the *DADA2* package (Callahan et al., 2016) within R. Reads were truncated to 228 and 230 bp and filtered with a maxEE (maximum number of “expected errors”) of 2 and 4 for forward and reverse reads, respectively (reads not reaching this threshold were discarded). *DADA2* constructs a parametric error matrix (based on the first 10^8^ bps in the dataset), the samples are dereplicated and sequence variants for the forward and reverse reads are inferred based on the derived error profiles from the samples. Singletons observed in the inference step are discarded. Subsequently, paired-end reads were merged with a maximum mismatch of 1 bp and a required minimum overlap of 10 bp. Forward and reverse reads, which did not merge were not included in further analysis. Chimeras were removed using the function removeBimeraDenovo. The resulting chimera-checked, merged amplicon sequence variants (ASV) were used for taxonomic classification using the SILVA v132 database (Pruesse et al., 2007) within the *DADA2* package, which is based on the rdp classifier (Wang et al., 2007) with a bootstrap of 50. The results were parsed into a table using the *phyloseq* package (Mcmurdie and Holmes, 2013), and reads assigned as eukaryotes, chloroplasts and mitochondria were removed. Negative controls were assessed and the sum of reads from contaminating ASVs was subtracted from the samples. Samples were subsampled to an even depth of 9,500 sequences for comparison.

##### Spatial study

Phylogenetically annotated 16S rRNA sequences were used to characterize bacterial community composition of each tissue type at the family level. Donut charts were generated using the package *ggplot2* (Wickham, 2016) in R based on the average relative abundance of sequence reads attributed to a given bacterial family within each tissue type and for each site. A two-way permutational multivariate analysis of variance (PERMANOVA; Anderson, 2001) was used to examine the potential differences in community composition as a function of tissue types (digestive gland, siphon or cockle) and sampling sites, assessed at the ASV level. PERMANOVA was run on triangular similarity matrices derived from the fourth root transformed data calculating the Bray-Curtis. Visualization of the observed patterns was obtained by means of a principle coordinates analysis (PCoA) using a Bray-Curtis similarity matrix. Alpha diversity numbers were calculated using the estimate_richness script in R. Differences in alpha diversity between tissues were assessed by an analysis of variance (ANOVA) for the “Observed” diversity index. Comparative analysis of the bacterial communities from sites with low *P. australis* TTX concentrations (Bleinhem, Marahau and Riverton) with those from Hokianga Harbor which has *P. australis* with high TTX was undertaken to identify potential members of the bacterial community correlated with changes in TTX concentrations. The second replicate for the Hokianga siphons did not contain enough reads and was lost after rarefaction. The bacterial communities from the digestive glands of both *P. australis* and *A. stutchburyi* were compared using *phyloseq*. Relationships between TTX concentrations and the 20 most abundant ASVs in the 20 most abundant phyla were determined using linear regressions.

##### Temporal study

The core bacterial communities of digestive glands and siphons from *P. australis* over a one-year period was identified using the *microbiome* package in R (Lahti et al., 2017). ASVs present in at least 70% of samples were chosen as members of the core bacterial communities. A two-way PERMANOVA was used to identify potential differences in community composition as a function of organs and sampling months, assessed at the ASV level. PERMANOVA was run on triangular similarity matrices derived from the fourth root transformed data calculating the Bray-Curtis. Alpha diversity numbers were calculated using the estimate_richness script in R. Differences in alpha diversity between tissues were assessed by an analysis of variance (ANOVA) for the “Observed” diversity index Visualization of the observed patterns was plotted with principle coordinates analysis using the Bray-Curtis similarity matrix. The core bacterial communities of the digestive glands from *A. stutchburyi* was also identified using the *microbiome* package.

## Results

### Tetrodotoxin Analysis

#### Spatial Study

**FIGURE 1:**
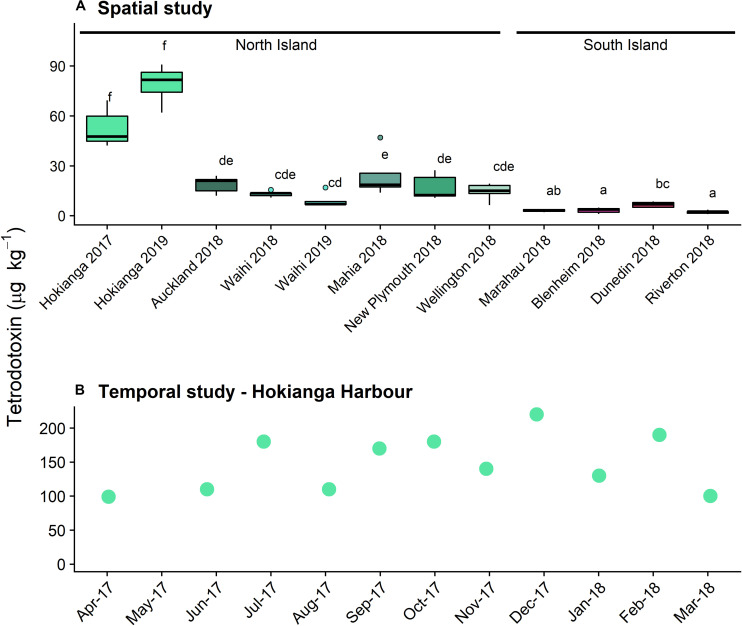
Tetrodotoxin concentrations in populations of *Paphies australis*, **(A)** collected around the New Zealand coastline, determined using liquid chromatography-mass spectrometry (LC/MS; *n* = 5). Solid black line shows median, box shows 1st and 3rd quartiles, whiskers extend to the last data point within 1.5 times the inter-quartile range. Dots outside the whiskers are considered as outliers, defined as beyond 1.5× interquartile range. Different letters indicate where significant differences occur between sites (one-way ANOVA with Tukey’s HSD *post-hoc* test, *p* < 0.001). Sites are ordered by increasing latitude for each island. Data from 2018 and earlier was previously reported in Biessy et al. (2019b). **(B)** Tetrodotoxin concentrations in populations of *P. australis* collected from the Hokianga Harbor site over a one-year period, determined using LCMS (*n* = 1 pooled sample).

Tetrodotoxin was detected in *P. australis* from sites all around New Zealand (Figure 1A) with TTX as the main congener (>99%) in all samples. A Levene’s test showed that the degree of variance did not differ among sites (*F* = 0.59). One-way ANOVA showed a significant difference between TTX concentrations among all sites (*F* = 51.6, *p* < 0.001), with a Tukey’s HSD *post-hoc* test identifying a complex overlap between sites (Figure 1A). The highest concentrations were measured in *P. australis* from the northernmost site, the Hokianga Harbor from both years tested. Median toxin concentrations in *P. australis* from the South Island sites were significantly lower than the North Island sites. No TTX (<3 μg kg^–1^) was detected in the *A. stutchburyi* samples from the Hokianga Harbor site.

#### Temporal Study

Tetrodotoxin was detected in all *P. australis* collected monthly from the Hokianga Harbor between April 2017 and March 2018 (Figure 1B). The lowest concentration was 99 μg k^–1^ in April 2017 and the highest was 220 μg kg^–1^ in December 2017 and the toxin averaged 150 μg kg^–1^ over the year of sampling. Only one sample was extracted for toxin analysis per time point and statistical analyses were not possible. The differences for the Hokianga Harbor toxin concentration between the spatial and temporal study are likely due to the minor methodological differences in toxin extraction.

### Metabarcoding

#### Spatial Study

Across the 64 samples, encompassing the 10 sites and two tissues, the 16S rRNA gene sequencing yielded 6,865,560 reads sequences that resulted in a total of 17,188 distinct ASVs after processing and rarefaction. The number of reads for each sample at the various processing step is provided in Supplementary Table S1.

**FIGURE 2:**
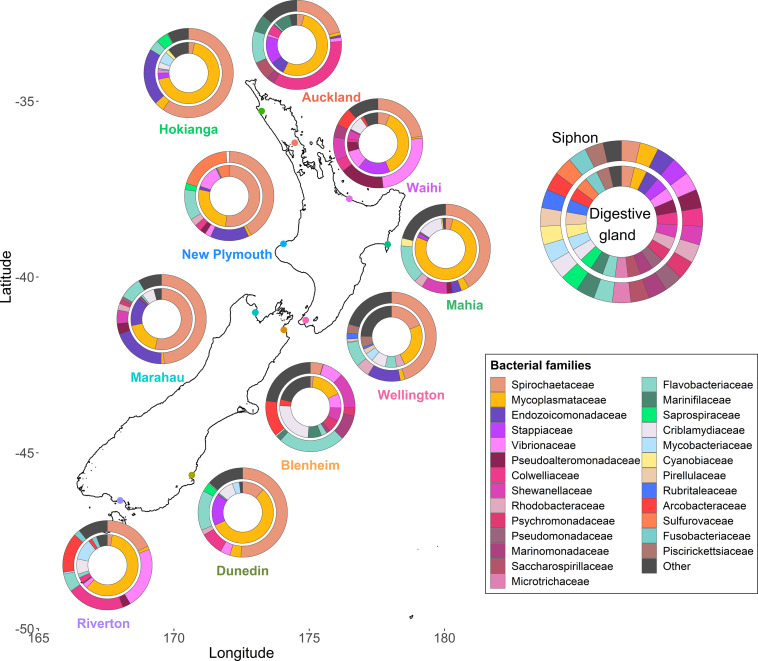
Bacterial community composition at the family level from the digestive glands and siphons of the New Zealand endemic clam *Paphies australis* collected from ten sites across the north and south islands. The relative abundance of the ten most abundant families in terms of sequence number for each site and organ are shown. Family are represented by different colors but the phylum Proteobacteria is represented by the purple-pink hues and the Bacteroidetes are represented by the green hues.

Species diversity significantly differed amongst the ten sites around New Zealand (*p* < 0.001, *F* = 5.9) but no differences between the two islands were observed (Supplementary Figure 2). The siphons had bacterial communities with significantly higher alpha diversity than the digestive glands (*p* < 0.001, *F* = 65.8). When examining the most abundant (top ten) families in term of read numbers (Figure 2), Spirochaetaceae (ranging from 4 to 60% across all samples) and Mycoplasmataceae (16–78%) accounted for the highest proportions of reads in the siphons and digestive glands, respectively, for both islands. Proteobacteria (purple and pink hues; Figure 2) were present at all sites and in both organs. Bacteroidetes (green hues, Figure 2) were present at all sites, except Waihi, and in higher abundances in the siphons. Cyanobiaceae were detected in two sites, Hokianga and Mahia (Figure 2).

**FIGURE 3:**
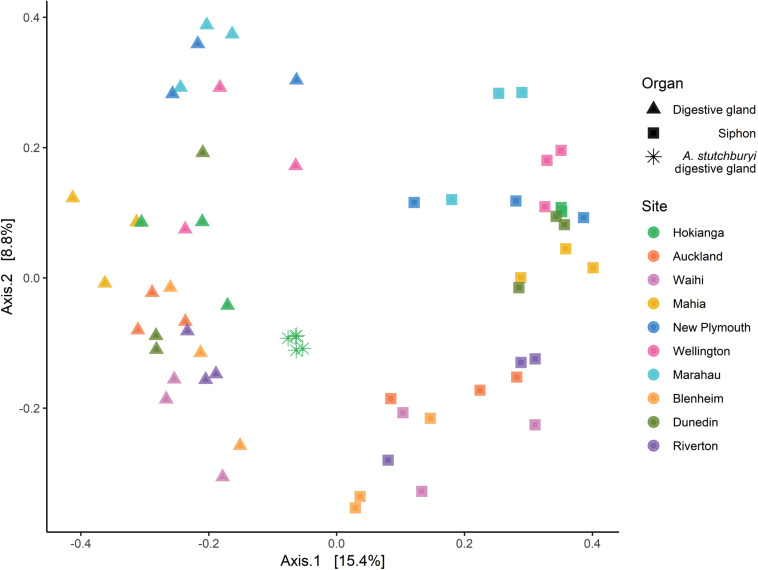
Principal Coordinates Analysis (PCoA) based on Bray-Curtis dissimilarities with 999 permutations of bacterial community composition of *Paphies australis* organs (unless indicated otherwise) at the Amplicon Sequence Variant level from ten different sites around New Zealand. For each axis, in square brackets, the percent of variation explained was reported. Sites are ordered by increasing latitude for New Zealand. *A. stutchburyi* = *Austrovenus stutchburyi*.

Multidimensional scaling based on Bray-Curtis similarities between bacterial communities (using ASVs), showed a clear separation between the two organs from *P. australis* and *A. stutchburyi* digestive gland (Figure 3). Comparison of bacterial communities across tissues and sites showed that the bacterial communities structure had a significant interaction term (PERMANOVA, *p* < 0.001, *F* = 1.61), suggesting inconsistent differences amongst the sites between organs.

**FIGURE 4:**
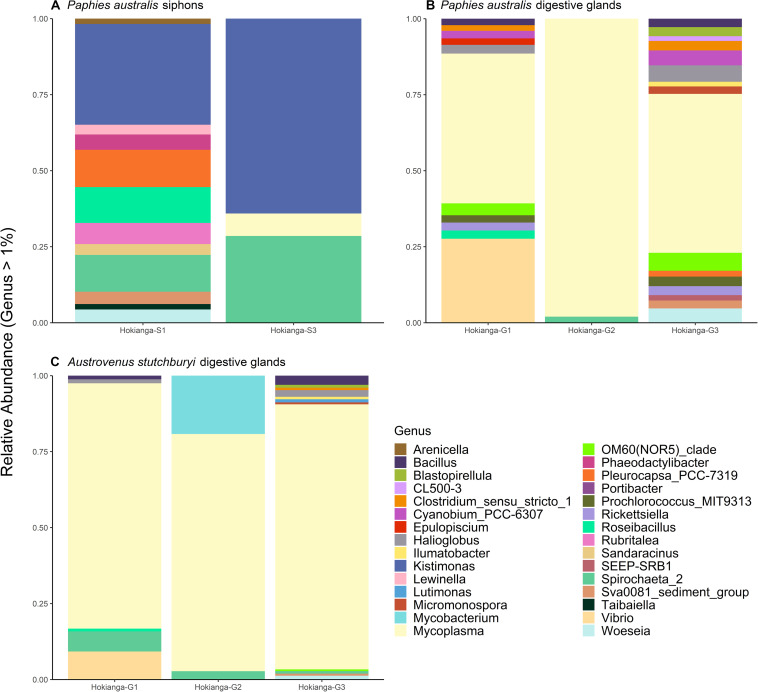
Stacked barplots showing the relative abundance of bacterial 16S ribosomal RNA sequences, at the genus level (>1% of total abundance), that are present in tetrodotoxin (TTX)-bearing *Paphies australis* from the Hokianga Harbor samples but not in tissue samples from sites with low TTX. **(A,B)** show communities present in *P. australis’* siphons and digestive glands, respectively, at the Hokianga site but not in similar tissues from the three lowest TTX sites. **(C)** shows relative abundance of bacteria communities present in TTX-bearing *P. australis’ digestive glands* and the non-toxic *Austrovenus stutchburyi’s* digestive gland from the same bed in the Hokianga Harbor. S1: Siphon replicate 1, S3: Siphon replicate 3, G1: digestive gland replicate 1, G2: digestive gland replicate 2, G3: digestive gland replicate 3.

The bacterial community was compared between the pooled sites with the lowest TTX concentrations (Blenheim, Marahau, and Riverton) and the ones from the Hokianga Harbor containing the highest toxin concentration. The most abundant bacterial genera in *P. australis*’ siphons from the Hokianga Harbor (Figure 4A) that were not present in low TTX sites were *Kistimonas* and *Spirochaeta_2* followed by *Pleurocapsa* and *Roseibacillus*. For the digestive glands, *Mycoplasma* was the main genus remaining in the *P. australis* from the Hokianga Harbor after ASVs from the low TTX sites had been subtracted (Figure 4B). Other genera present included *Bacillus*, *Clostridium*, *Prochlorococcus* and *Vibrio*. Finally, when comparing the bacterial communities present in the digestive glands of the TTX-bearing *P. australis* to those in the non-toxic *A. stutchburyi*, *Mycoplasma* was also the most abundant genus remaining. *Bacillus*, *Mycobacterium*, *Spirochaeta*, and *Vibrio* were also present (Figure 4C).

The results from the linear regression analysis identified 17 ASV that were significantly and positively correlated to TTX concentrations, however, all relationships were weak (*R*^2^ < 0.35; Supplementary Table S2). The phyla present were: Verrucomicrobia (4 ASVs from the *Rubritaleaceae* family), Tenericutes (4 ASVs from the *Mycoplasmataceae* family), Acidobacteria (2 ASVs), Cyanobacteria (2 ASVs including the genus *Synechococcu*s_CC9902), Marinimicrobia (2 ASVs), and one ASVs from each Firmicutes (*Romboutsia* genus), Planctomycetes and Proteobacteria phyla.

#### Temporal Study

##### Bacterial composition

Bacterial DNA was isolated from *P. australis* siphons and digestive glands every month for 11 months (*n* = 3 per month), totaling 62 samples after rarefaction and contamination removal. The 16S rRNA gene sequencing yielded 589,000 reads that resulted in 14,782 distinct ASVs. The number of reads for each sample at the various processing steps is provided in Supplementary Table S1.

**FIGURE 5:**
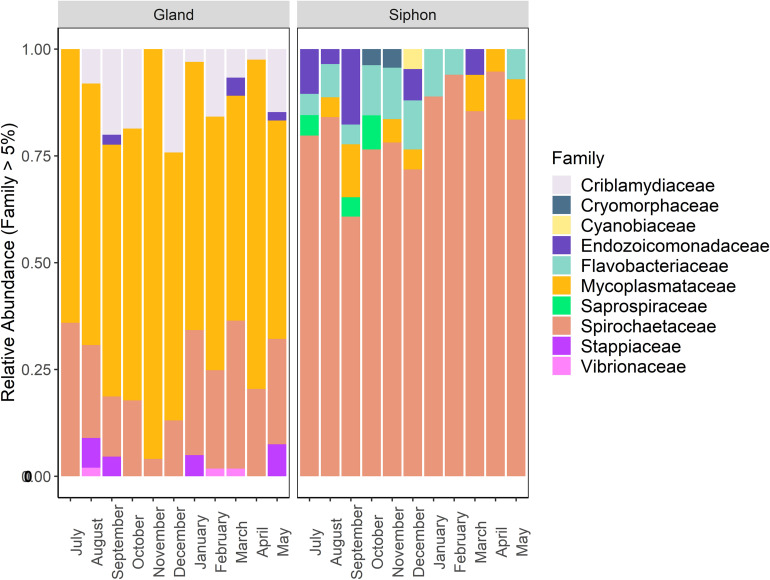
Stacked bar plot showing the relative abundance of bacterial 16S ribosomal RNA sequences at the family level (contributing to >5% of total abundance) in *Paphies australis* digestive glands and siphons from the Hokianga Harbor (Northland, New Zealand), sampled every month from July 2017 to May 2018.

Taxonomic community composition of the dominant families (abundance > 5%) by tissue type revealed that similar numbers were present in both *P. australis’* tissues (Figure 5). There were high relative abundances of Mycoplasmataceae (65–95%) and Spirochaetaceae (60–90%) in the digestive glands and siphons, respectively. In the siphons, Flavobacteriaceae (0–14%) and Mycoplasmataceae (0–12%) were also substantial components of the community occurring in samples from most months. Endozoicomonadaceae (0–18%) were also present half of the year, mostly in winter (July–September). In the digestive glands, Spirochaetaceae (4–37%) and Criblamydiaceae (0–25%) were present in samples from the majority of the sampling period. Stappiaceae (0–8%), Endozoicomonadaceae (0–4%), and Vibrionaceae (0–2%; Figure 5) were less frequently observed in the digestive gland communities (Figure 5).

**FIGURE 6:**
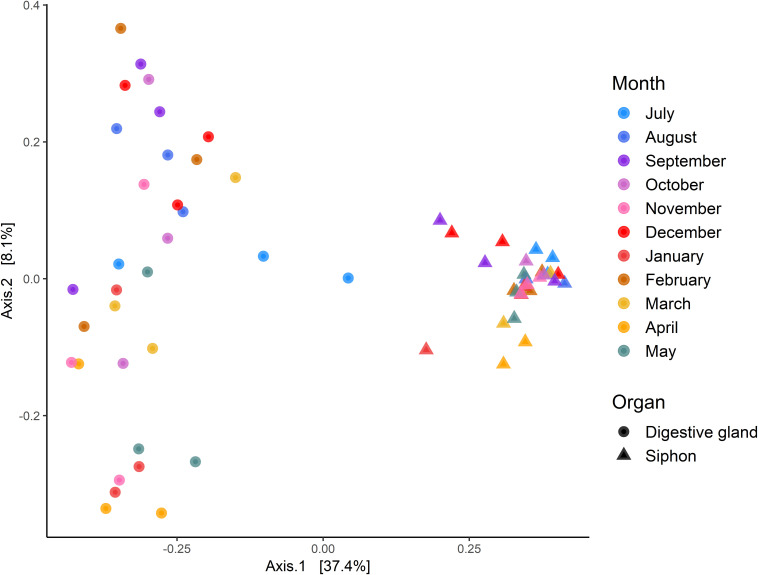
Principal Coordinates Analysis (PCoA) based on Bray-Curtis dissimilarities with 999 permutations of bacterial community composition of *Paphies australis’* organs at the Amplicon Sequence Variant level, sampled every month from July 2017 to May 2018 from the Hokianga Harbor (Northland, New Zealand). For each axis, in square brackets, the percent of variation explained is reported.

Multidimensional scaling based on Bray-Curtis similarities between bacterial communities (using ASVs), showed a clear separation between the two organs (Figure 6). Analysis of overall bacterial community structure revealed a significant difference between siphons and digestive glands (PERMANOVA: *p* < 0.0001, *F* = 11.5). No significant interaction was found between sampling months and tissue type (PERMANOVA, *p* = 0.12, *F* = 1.26). The digestive glands did not display a seasonal pattern in species diversity but had a significantly lower diversity than the siphons (*p* < 0.001, *F* = 181.85; Figure 6). In contrast, the siphons had a higher diversity in warmer months (October to January; Supplementary Figure S3).

##### Core bacterial communities

A total of 38 and 74 bacterial ASVs present in at least 70% of all samples were identified as members of the core bacterial communities of the *P. australis’* digestive glands and siphons, respectively (Tables 1, 2). Mycoplasmatales (15 ASVs total) was the most dominant order in the digestive gland bacterial communities, followed by Spirochaetales (9 ASVs total). Many more orders were present in the core bacterial communities of the siphons from *P. australis* with the Flavobacteriales the most abundant (12 ASVs total), followed closely by Chitinophagales (7 ASVs), Microtrichales (7 ASVs) and Steroidobacterales (5 ASVs; Table 2). Only 12 ASVs were present in the core bacterial communities of *A. stutchburyi*’s digestive glands (Supplementary Table S3): Clostridiales was the most dominant order (4 ASVs), followed by Mycoplasmatales (3 ASVs).

**TABLE 1:** Summary of bacterial amplicon sequence variants (ASV) corresponding to the core bacterial communities of *Paphies australis’* digestive glands, define as present in at least 70% of all samples.

Phylum	Class	Order	Family	Genus	Number of ASVs
Tenericutes	Mollicutes	Mycoplasmatales	Mycoplasmataceae	***Mycoplasma***	14
Spirochaetes	Spirochaetia	Spirochaetales	Spirochaetaceae	***Spirochaeta_2***	6
Spirochaetes	Spirochaetia	Spirochaetales	Spirochaetaceae	Unclass.	3
Actinobacteria	Actinobacteria	Corynebacteriales	Mycobacteriaceae	*Mycobacterium*	1
Bacteroidetes	Bacteroidia	Chitinophagales	Saprospiraceae	Unclass.	1
Chlamydiae	Chlamydiae	Chlamydiales	Criblamydiaceae	Unclass.	1
Cyanobacteria	Oxyphotobacteria	Synechococcales	Cyanobiaceae	*Synechococcus_CC9902*	1
Firmicutes	Bacilli	Bacillales	Bacillaceae	*Bacillus*	1
Firmicutes	Clostridia	Clostridiales	Lachnospiraceae	*Epulopiscium*	1
Firmicutes	Clostridia	Clostridiales	Peptostreptococcaceae	*Romboutsia*	1
Proteobacteria	Alphaproteobacteria	Rhizobiales	Stappiaceae	Unclass.	1
Proteobacteria	Unclass.	Unclass.	Unclass.	Unclass.	1
Proteobacteria	Alphaproteobacteria	Rhizobiales	Unclass.	Unclass.	1
Tenericutes	Mollicutes	Mycoplasmatales	Mycoplasmataceae	Unclass.	1

*In bold are the genera detected in more than 90% of all samples. Unclass. = unclassified.*

**TABLE 2:** Summary of bacterial amplicon sequence variants (ASVs) corresponding to the core bacterial communities of the clam *Paphies australis’* siphons, present in at least 70% of all samples.

Phylum	Class	Order	Family	Genus	ASVs
Bacteroidetes	Bacteroidia	Chitinophagales	Saprospiraceae	Unclass.	7
Proteobacteria	Gammaproteobacteria	Steroidobacterales	Woeseiaceae	*Woeseia*	5
Actinobacteria	Acidimicrobiia	Microtrichales	Microtrichaceae	*Sva0996_marine_group*	4
Bacteroidetes	Bacteroidia	Flavobacteriales	Flavobacteriaceae	*Aquibacter*	4
Actinobacteria	Acidimicrobiia	Microtrichales	Ilumatobacteraceae	*Ilumatobacter*	3
Proteobacteria	Gammaproteobacteria	Unclass.	Unclass.	Unclass.	3
Spirochaetes	Spirochaetia	Spirochaetales	Spirochaetaceae	***Spirochaeta_2***	3
Bacteroidetes	Bacteroidia	Flavobacteriales	Flavobacteriaceae	*Muriicola*	2
Actinobacteria	Acidimicrobiia	Actinomarinales	Unclass.	Unclass.	2
Cyanobacteria	Oxyphotobacteria	Synechococcales	Cyanobiaceae	***Cyanobium_PCC-6307***	2
Cyanobacteria	Oxyphotobacteria	Synechococcales	Cyanobiaceae	***Synechococcus_CC9902***	2
Proteobacteria	Deltaproteobacteria	Desulfobacterales	Desulfobulbaceae	Unclass.	2
Proteobacteria	Gammaproteobacteria	Cellvibrionales	Halieaceae	*Halioglobus*	2
Proteobacteria	Gammaproteobacteria	Gammaproteobacteria_Incertae_Sedis	Unknown_Family	Unclass.	2
Proteobacteria	Gammaproteobacteria	UBA10353_marine_group	Unclass.	Unclass.	2
Spirochaetes	Spirochaetia	Spirochaetales	Spirochaetaceae	Unclass.	2
Tenericutes	Mollicutes	Mycoplasmatales	Mycoplasmataceae	*Mycoplasma*	2
Acidobacteria	Thermoanaerobaculia	Thermoanaerobaculales	Thermoanaerobaculaceae	*Subgroup_10*	1
Bacteroidetes	Bacteroidia	Flavobacteriales	Flavobacteriaceae	*Eudoraea*	1
Bacteroidetes	Bacteroidia	Flavobacteriales	Flavobacteriaceae	*Lutimonas*	1
Bacteroidetes	Bacteroidia	Flavobacteriales	Flavobacteriaceae	*Polaribacter_4*	1
Bacteroidetes	Bacteroidia	Flavobacteriales	Flavobacteriaceae	*Robiginitalea*	1
Bacteroidetes	Bacteroidia	Flavobacteriales	Flavobacteriaceae	*Winogradskyella*	1
Bacteroidetes	Bacteroidia	Flavobacteriales	Flavobacteriaceae	*Zeaxanthinibacter*	1
Cyanobacteria	Oxyphotobacteria	Leptolyngbyales	Leptolyngbyaceae	Unclass.	1
Cyanobacteria	Oxyphotobacteria	Synechococcales	Cyanobiaceae	*Prochlorococcus_MIT9313*	1
Proteobacteria	Alphaproteobacteria	Rhizobiales	Unclass.	Unclass.	1
Proteobacteria	Alphaproteobacteria	Rhizobiales	Rhizobiales_Incertae_Sedis	*Anderseniella*	1
Proteobacteria	Alphaproteobacteria	Rhodobacterales	Rhodobacteraceae	*Boseongicola*	1
Proteobacteria	Alphaproteobacteria	Rhodobacterales	Rhodobacteraceae	Unclass.	1
Proteobacteria	Alphaproteobacteria	Rhodobacterales	Rhodobacteraceae	*Silicimonas*	1
Proteobacteria	Gammaproteobacteria	Arenicellales	Arenicellaceae	Unclass.	1
Proteobacteria	Gammaproteobacteria	Oceanospirillales	Endozoicomonadaceae	*Kistimonas*	1

*In bold are the genera detected in more than 90% of all samples. Unclass. = unclassified.*

## Discussion

The endemic New Zealand clam *P. australis* is an excellent study organism to investigate the source of TTX. Individual clams are mostly stationary, and samples can be repeatedly collected from the same location over time, reducing the effects of seasons, tides and weather conditions. This study explored seasonal and spatial differences in bacterial communities’ present in the siphons and digestive glands of *P. australis* and aimed to identify any relationships with TTX-production. To achieve this, the bacterial communities were characterized using metabarcoding in *P. australis* sourced monthly from the Hokianga Harbor for 1 year and the bacterial communities of *P. australis* from 10 sites with varying TTX concentrations spanning the length of New Zealand were compared. Previous studies have shown that *P. australis* from the Hokianga Harbor contained relatively high levels of TTX and that toxin concentrations in clam populations were variable around the country (Biessy et al., 2019b).

The bacterial community present in the siphons was more diverse than the digestive glands, potentially due to being in direct contact with the external environment (Pierce and Ward, 2018). Previous studies on other bivalves have shown similar patterns with the gills of oysters *Crassostrea gigas* containing a higher diversity of bacteria than digestive glands (Hernández-Zárate and Olmos-Soto, 2006). Kueh and Chan (1985) also found that total bacterial counts were comparable between seawater and organs in contact with the external environment like the gills, mantle and adductor muscles of the oyster *C. gigas*. The most abundant phylum in the siphon was Spirochetes. Species from this phylum have previously been found associated with the crystalline style or stomach of bivalves (Gunnar et al., 2010) but are also free-living bacteria that are abundant in marine environments, explaining their abundance in the siphons of shellfish (Paster and Dewhirst, 2000). The bacterial communities of the digestive glands were dominated by ASVs from the genus *Mycoplasma*, a genus that is also consistently associated with bivalves, often in high abundances (Lokmer et al., 2016; Aceves et al., 2018; Pierce and Ward, 2018). Proteobacteria such as Vibrionaceae, Stappiaceae or Endozoicomonadaceae were also abundant in both organs, which corroborates previous studies of TTX-bearing bivalves, for example in the clams *Ruditapes philippinarum* (Milan et al., 2018).

Many bacterial genera have been reported as potential producers of TTX, the most common being *Bacillus*, *Pseudomonas*, *Aeromonas*, *Vibrio*, *Actinomyces*, and *Shewanella* (Pratheepa and Vasconcelos, 2013), and over 150 TTX-producing strains are described in the literature (Magarlamov et al., 2017; Katikou, 2019). With less than 1% of bacteria being culturable there are challenges with isolating, culturing and thus characterizing TTX producers and it is likely that many other strains may be TTX producers (Chau et al., 2011). Many marine bacteria are capable of producing toxic secondary metabolites including; cyanobacteria (Williamson et al., 2004; Mejean et al., 2010), *Actinomycetes* (Fiedler et al., 2005), and *Bacilli* (Teasdale et al., 2009). The development of molecular techniques, such as metabarcoding, have revolutionized environmental surveys and are increasingly used worldwide for biodiversity monitoring (Taberlet et al., 2012; Valentini et al., 2016; Stat et al., 2017). Metabarcoding has previously been used for detecting rare, toxin-producing dinoflagellates (Smith et al., 2017), to first report harmful algal species in new territories (Grzebyk et al., 2017) and to monitor toxic cyanobacterial blooms in freshwater reservoirs (Casero et al., 2019). Salvitti et al. (2017) used metabarcoding to investigate the diet and gut content of the TTX-containing sea slug *P. maculata* and observed that most TTX-bearing individuals contained Cnidarian and Annelids sequences, two phyla known to contain toxic species (Miyazawa and Noguchi, 2001; Salvitti et al., 2017). However, the bacterial content of the slug’s foregut was not analyzed and blocking primers to remove the background sequences of the sea slugs themselves were not used, reducing the number of taxa that could be identified (Vestheim and Jarman, 2008). Another study investigating the microbiome of TTX-containing marine nemertean *Cephalothrix simula* showed the prevalence of a large number of bacterial genera previously associated with TTX production including *Alteromonas*, *Vibrio* and *Pseudomonas* but could not conclusively identify a potential producer (Turner et al., 2018).

The present study comprized of a spatial and temporal component. When analyzing the spatial study data, we subtracted the bacterial ASVs from sites containing very low TTX concentrations (approx. limit of detection; 2–3 μg kg^−1^) from the Hokianga Harbor bacterial ASVs. In the siphons, the first genus of interest was *Phaeodactylibacter*, a gram-negative bacteria associated with the diatoms *Phaeodactylum* spp. (Chen et al., 2014) that have been shown to produce the neurotoxin non-protein amino acid β-methylamino-L-alanine (BMAA) (Réveillon et al., 2016). *Bacillus* (phylum: Firmicutes) was detected in the digestive glands from *P. australis* from high TTX sites and has previously been described as a potential TTX producer (Chau, 2013) and TTX was recently detected in the strain *Bacillus* sp. 1839 tested after 5 years of cultivation in the laboratory (Melnikova et al., 2019). However, the concentrations detected in the culture were low and would not likely account for the extremely high concentrations in higher trophic organisms. The last genus of interest in the differential analysis was *Vibrio*, which comprise more than 30% of all the suggested 150 TTX-producing strains reported in the literature (Turner et al., 2018). When the bacteria present in the digestive glands from the non-toxic *A. stutchburyi* were subtracted from those in the glands of toxic *P. australis*, the genera *Bacillus* and *Vibrio* were also present indicating that these taxa remain candidates for further investigation as potential TTX producers.

In the temporal study, the focus was on the core bacterial communities of TTX-bearing *P. australis* over an entire year. The core microbiome of a species is defined as the group of microbes present in all individuals regardless of the environment (Turnbaugh et al., 2007). Given that TTX was present in all samples in the temporal study at Hokianga Harbor, we hypothesized that the TTX producer would be present in the core bacterial communities of *P. australis.* The siphons and the digestive glands had some similarities: two genera, *Mycoplasma* and *Spirochaeta*, were present in both organs’ bacterial communities. *Spirochaeta* and *Mycoplasma* are common in the core bacterial communities of bivalves (Pierce and Ward, 2018) and were also present from all sites in the spatial study but have never been previously associated with toxin production. In the digestive glands, *Bacillus* was present in 70% of all samples.

When analyzing the spatial study data, three cyanobacterial genera remained when subtracting the bacterial ASVs from sites containing very low TTX concentrations from the Hokianga Harbor bacterial ASVs. The first was *Pleurocapsa* strain PCC-7319, a cyanobacterium that was found to be abundant in a toxic bacterial mat, although the toxin was not identified (Aboal et al., 2002). Two other cyanobacterial genera, *Cyanobium* strain PCC-6307 and *Prochlorococcus* strain MIT-9313, were also detected. *Cyanobium* have been shown to produce toxins similar to microcystins (Jakubowska and Szeląg-Wasielewska, 2015) and one strain (in addition to other cyanobacteria) was toxic to brine shrimp, but the analysis did not reveal the production of known toxins (Frazão et al., 2010). *Prochlorococcus* is one of the smallest and most abundant photosynthetic organisms in the ocean (Partensky et al., 1999) and at least one strain has been shown to produce BMAA (Jakubowska and Szeląg-Wasielewska, 2015). *Prochlorococcus* and *Cyanobium* are picocyanobacteria (i.e., have a cell size between 0.2 and 2 μm) and are common in freshwater and marine ecosystems but remain poorly studied due to their small size and difficulty to isolate (Jakubowska and Szeląg-Wasielewska, 2015). Cyanobacterial ecotoxicology is particularly well documented in freshwater habitats where they have been found to produce a wide range of toxins (Jakubowska and Szeląg-Wasielewska, 2015; Rastogi et al., 2015; Cirés et al., 2017) including saxitoxin. Saxitoxin is a potent neurotoxin produced by cyanobacteria and marine dinoflagellates and is known to exert the same toxic effect as TTX through an interaction with voltage gated sodium channels resulting in inhibition of neuromuscular transmission (Narahashi, 1988). Both toxins are active on the α-subunit of the sodium channels (Walker et al., 2012) and have been detected simultaneously in several aquatic species (Bane et al., 2014). However, toxicological studies on cyanobacteria have not been performed to the same extent in marine environments (Frazão et al., 2010), and while no specific links have previously been reported between cyanobacteria and TTX, they are an interesting new lead for future studies with the aim to identify TTX-producing bacterial species.

The unicellular cyanobacterial genus *Synechococcus* was significantly correlated to higher TTX concentrations in the spatial study and was also present in the bacterial communities of both *P. australis*’ organs in the temporal study. Marine *Synechococcus* are abundant throughout the world’s oceans and are significant primary producers in coastal environments but very little is known about their toxic effects on other marine organisms (Hamilton et al., 2014). Some strains produce substances with neurotoxic effects in mice and negative effects on invertebrates (Martins et al., 2005, 2007). Although no toxin production has been associated with the strain CC9902 (the strain whose 16S rRNA sequences were most closely related to the ASV in this study; Genbank accession number CP000097.1), it has been shown to alter fish behavior after a 3-day exposure (Hamilton et al., 2014). More toxicological studies should be undertaken on *Synechococcus* using analytical methods. When investigating the core bacterial communities of the siphons, *Cyanobium* PCC-6307 and *Synechococcus* CC9902 were again present in 90% of all samples and *Prochlorococcus* MIT9313 in 70% of all samples, strengthening the hypothesis that cyanobacteria (especially picocyanobacteria) could be responsible for the presence of TTX in *P. australis*. This is further reinforced by the fact that no cyanobacteria were present in the core bacterial communities of the non-TTX bearing *A. stutchburyi*.

## Conclusion and Future Research

This is the first study to evaluate the bacterial communities of TTX-bearing and non-TTX bearing bivalves using high-throughput sequencing. Our results from both spatial and temporal studies correlate with some previous hypotheses that *Vibrio* and *Bacillus* could be responsible for the source of TTX in the New Zealand endemic clams *P. australis.* This study highlights that further investigation of marine cyanobacteria as potential TTX producers is warranted, especially *Synechococcus, Cyanobium*, and *Prochlorococcus* that were present in both spatial and temporal studies. Culturing, isolating and testing these bacterial species, in particular picocyanobacteria, for toxin production would be a logical next step in the search to identify the source of TTX in marine organisms. It has recently been shown that DNA from toxic flatworms were detected in the intestinal content of juvenile pufferfish (Okabe et al., 2019). So alternatively, it is also feasible that *P. australis* could source TTX from ingesting larvae, eggs, or pieces of the highly toxic sea slug *P. maculata* and flatworm *Stylochoplana* sp., which are common throughout New Zealand, Japan and Europe (Miyazawa et al., 1987; Salvitti et al., 2015; Turner et al., 2018). Other eukaryotes communities, such as dinoflagellates, should also be investigated as many of them produce other marine biotoxins. Molecular techniques such as qPCR, droplet digital PCR or metabarcoding with the use of blocking primers could be used to assess whether these toxic species are present in the digestive system of *P. australis*.

## Data Availability Statement

The datasets presented in this study can be found in online repositories. The names of the repository/repositories and accession number(s) can be found in the article/ Supplementary Material.

## Author Contributions

LB, KS, IH, and SW conceived and designed the experiments. LB performed the experiments. LB and JP undertook the metabarcoding. KS, JP, and SW helped with the data interpretation and experiments. All authors contributed to data analysis and the editing and writing of the manuscript.

## Conflict of Interest

The authors declare that the research was conducted in the absence of any commercial or financial relationships that could be construed as a potential conflict of interest.
