# Supplementary material for: Seasonal and Spatial Variations in Bacterial Communities From Tetrodotoxin-Bearing and Non-tetrodotoxin-Bearing Clams
Source: Front Microbiol. 2020 Aug 5;11:1860. doi: 10.3389/fmicb.2020.01860 (PMC7419435; doi:10.3389/fmicb.2020.01860)
Supplement: Supplementary file 1 [file Data_Sheet_1.docx]

Supplementary Material

## Supplementary Figures


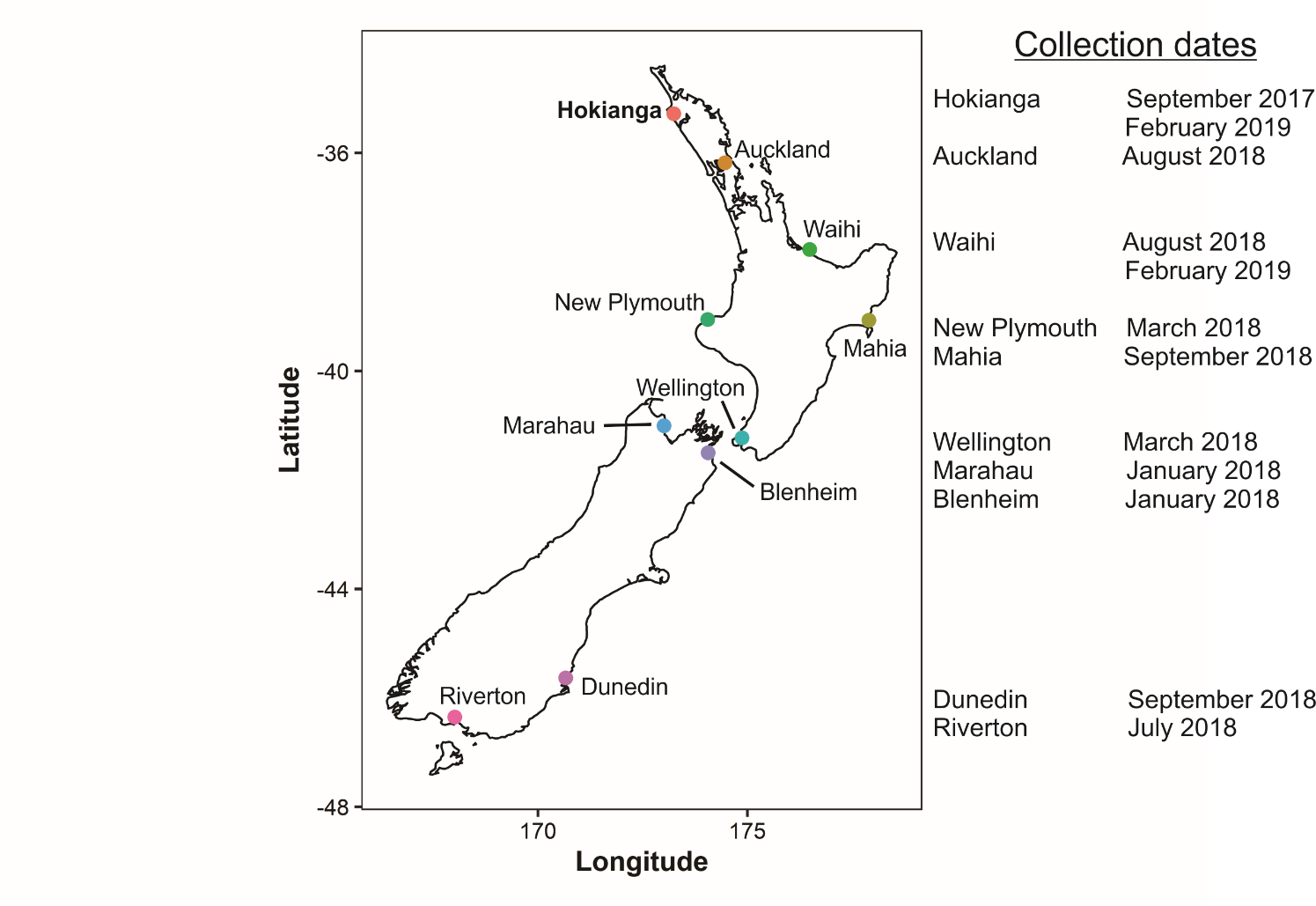


**Supplementary Figure 1**. *Paphies australis* collection dates and locations around New Zealand.

**
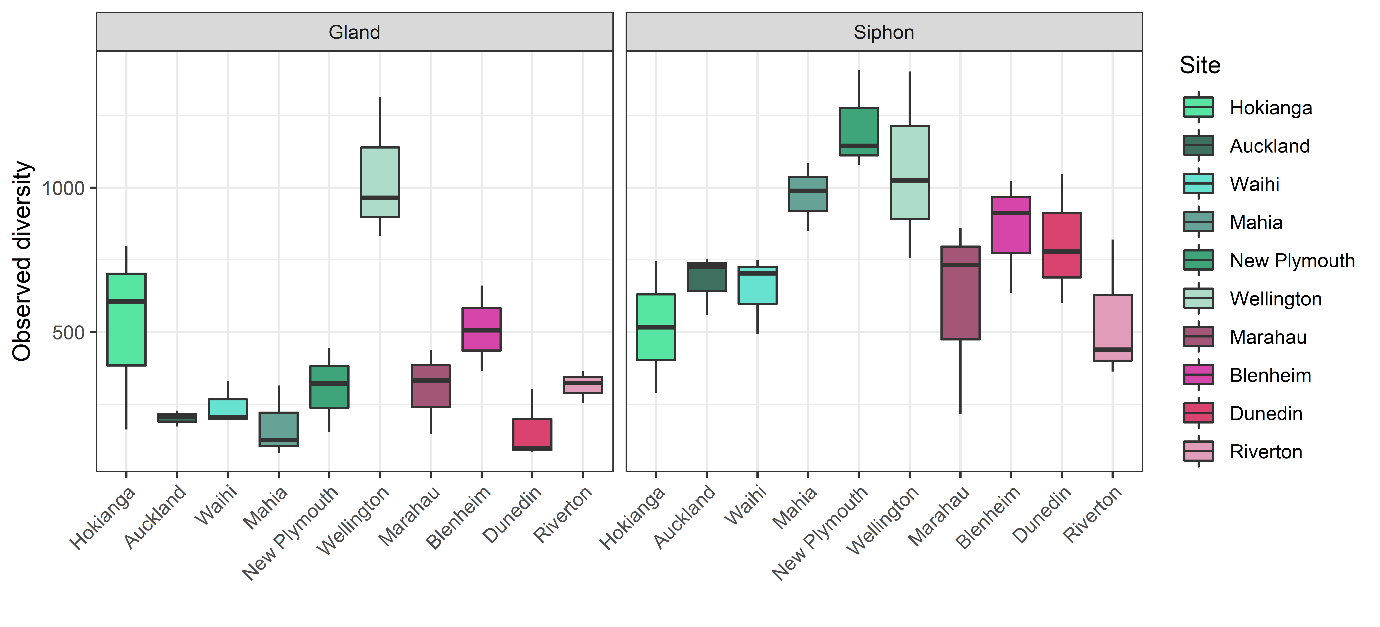
Supplementary Figure 2.** Boxplot displaying differences in community diversity between *Paphies australis* digestive glands and siphons from ten sites around New Zealand. Boxes display the first and third quartile spread of the data, with the line in the box indicating the median, the whiskers denoting the minimum and maximum values and the dots as outliers of the data. Sites are ordered by increasing latitude for each Island, the green colours represent the sites from the North Island and the pinks represent the sites of the South Island


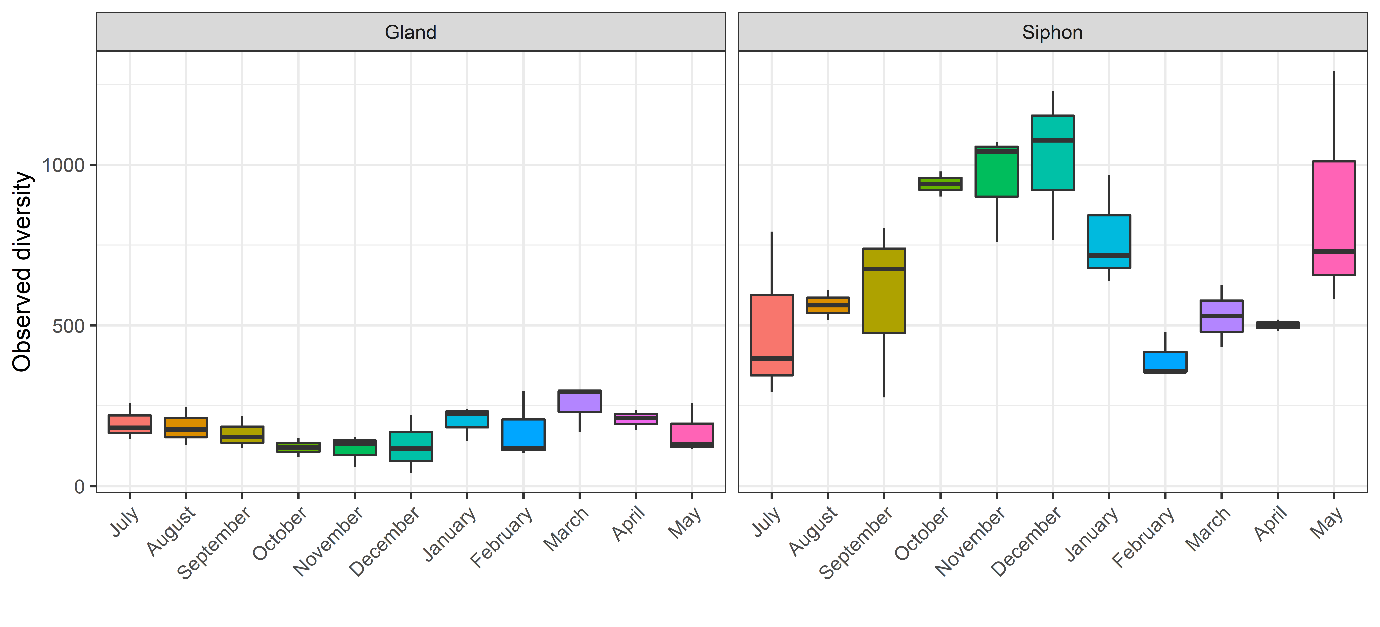


**Supplementary Figure 3.** Boxplot displaying differences in community diversity between *Paphies australis* digestive glands and siphons from the Hokianga Harbour (Northland, New Zealand), sampled every month from July 2017 to May 2018. Boxes display the first and third quartile spread of the data, with the line in the box indicating the median, the whiskers denoting the minimum and maximum values and the dots as outliers of the data. Sites are ordered by increasing latitude for each Island, the green colours represent the sites from the North Island and the pinks represent the sites of the South Island.

## Supplementary Tables

**Table 1.** Number of reads at each stage of the metabarcoding processing using the *dada2* package in R software.

| **Samples** | **Input** | **Filtered** | **Forward denoised** | **Reverse denoised** | **Merged** | **Chimeras removed** |
| --- | --- | --- | --- | --- | --- | --- |
| **April18-G2** | 402415 | 370916 | 369613 | 369551 | 364324 | 304332 |
| **April18-G3** | 456801 | 420010 | 418675 | 418406 | 411425 | 379906 |
| **April18-S1** | 84390 | 76181 | 74379 | 74502 | 70069 | 65958 |
| **April18-S2** | 609 | 491 | 470 | 455 | 424 | 418 |
| **April18-S3** | 136802 | 124010 | 121887 | 121864 | 116980 | 108889 |
| **Aug17-G1** | 142467 | 129908 | 128719 | 128933 | 125250 | 114408 |
| **Aug17-G2** | 192550 | 175944 | 175207 | 175000 | 171387 | 156057 |
| **Aug17-G3** | 245615 | 225159 | 223857 | 223849 | 218680 | 192643 |
| **Aug17-S1** | 54391 | 47958 | 46395 | 46377 | 42466 | 40350 |
| **Aug17-S2** | 80573 | 71956 | 69830 | 69914 | 64516 | 61181 |
| **Aug17-S3** | 127 | 57 | 45 | 38 | 25 | 25 |
| **Dec17-G1** | 464443 | 418910 | 417817 | 417210 | 412368 | 385845 |
| **Dec17-G2** | 12967 | 11855 | 11784 | 11719 | 11228 | 10387 |
| **Dec17-G3** | 26469 | 24299 | 24004 | 24021 | 22787 | 20250 |
| **Dec17-S1** | 179206 | 153956 | 149790 | 150422 | 139691 | 131646 |
| **Dec17-S2** | 145740 | 129256 | 126457 | 126713 | 119040 | 112163 |
| **Dec17-S3** | 143320 | 122732 | 118187 | 118910 | 107456 | 99504 |
| **Feb18-G1** | 123333 | 112319 | 111885 | 111703 | 110114 | 106378 |
| **Feb18-G2** | 372436 | 342205 | 340537 | 340635 | 335554 | 310237 |
| **Feb18-G3** | 151964 | 141476 | 140739 | 140801 | 137827 | 117756 |
| **Feb18-S1** | 37619 | 33854 | 33019 | 33018 | 31470 | 30207 |
| **Feb18-S2** | 47913 | 43357 | 42194 | 42207 | 39427 | 37505 |
| **Feb18-S3** | 96278 | 86667 | 85753 | 85580 | 82645 | 79104 |
| **Jan18-G1** | 115977 | 107047 | 106608 | 106302 | 104118 | 93867 |
| **Jan18-G2** | 74626 | 67946 | 66986 | 67044 | 63948 | 55519 |
| **Jan18-G3** | 69367 | 63857 | 63063 | 62918 | 60426 | 57298 |
| **Jan18-S1** | 56661 | 47614 | 45688 | 45878 | 40631 | 38537 |
| **Jan18-S2** | 59396 | 51560 | 48961 | 49292 | 43138 | 40705 |
| **Jan18-S3** | 58980 | 51486 | 49851 | 49982 | 45786 | 42897 |
| **July17-G1** | 41820 | 36173 | 34162 | 34233 | 28996 | 27089 |
| **July17-G2** | 56839 | 52987 | 52577 | 52466 | 51138 | 48060 |
| **July17-G3** | 35419 | 32604 | 32197 | 32187 | 31316 | 30202 |
| **July17-S1** | 134564 | 122730 | 121986 | 121896 | 119160 | 111815 |
| **July17-S2** | 32115 | 28882 | 28157 | 28087 | 26232 | 24317 |
| **July17-S3** | 36547 | 32666 | 31469 | 31566 | 28996 | 27596 |
| **March18-G1** | 311531 | 287986 | 286963 | 286494 | 281006 | 247740 |
| **March18-G2** | 245257 | 225518 | 224058 | 224091 | 218773 | 191633 |
| **March18-G3** | 107412 | 98312 | 97106 | 97299 | 93461 | 83629 |
| **March18-S1** | 173654 | 155414 | 154136 | 154265 | 150839 | 141744 |
| **March18-S2** | 624 | 368 | 351 | 309 | 258 | 244 |
| **March18-S3** | 180853 | 164346 | 162590 | 162973 | 158041 | 151230 |
| **May18-G1** | 210340 | 194349 | 193769 | 193539 | 191215 | 167539 |
| **May18-G2** | 287881 | 261751 | 260518 | 260511 | 255035 | 239101 |
| **May18-G3** | 60041 | 56318 | 56005 | 56017 | 54810 | 49799 |
| **May18-S1** | 315043 | 277090 | 270939 | 271843 | 255192 | 243560 |
| **May18-S2** | 138896 | 122444 | 120039 | 120413 | 114203 | 108128 |
| **May18-S3** | 139270 | 125114 | 123144 | 123383 | 117967 | 111928 |
| **Nov17-G1** | 278102 | 258715 | 257808 | 257393 | 253018 | 219268 |
| **Nov17-G2** | 134284 | 124604 | 123995 | 123864 | 121247 | 101680 |
| **Nov17-G3** | 97671 | 91828 | 91590 | 91415 | 90225 | 73930 |
| **Nov17-S1** | 159830 | 139678 | 136241 | 136276 | 126462 | 119485 |
| **Nov17-S2** | 152396 | 133827 | 130964 | 131065 | 123489 | 117565 |
| **Nov17-S3** | 196866 | 172648 | 168452 | 169107 | 158345 | 149753 |
| **Oct17-G1** | 87557 | 82314 | 81914 | 81816 | 79688 | 64663 |
| **Oct17-G2** | 245167 | 225911 | 224904 | 224692 | 221069 | 202556 |
| **Oct17-G3** | 215587 | 201727 | 201073 | 200768 | 197004 | 157782 |
| **Oct17-S1** | 363 | 228 | 185 | 169 | 117 | 117 |
| **Oct17-S2** | 57688 | 50275 | 47526 | 47826 | 40882 | 37530 |
| **Oct17-S3** | 81013 | 70167 | 66421 | 66683 | 57768 | 52146 |
| **PCR Negative** | 291 | 98 | 72 | 69 | 42 | 42 |
| **PCR Negative2** | 2305 | 1863 | 1843 | 1835 | 1801 | 1801 |
| **Sept17-G1** | 299437 | 276766 | 274446 | 274424 | 266953 | 221977 |
| **Sept17-G2** | 32831 | 30852 | 30520 | 30573 | 29272 | 25878 |
| **Sept17-G3** | 184652 | 168362 | 167320 | 167116 | 162772 | 145502 |
| **Sept17-S1** | 71205 | 62603 | 59558 | 60040 | 52728 | 46919 |
| **Sept17-S2** | 73018 | 64686 | 61942 | 61705 | 54848 | 50741 |
| **Sept17-S3** | 69946 | 63529 | 62044 | 62139 | 58465 | 53165 |
| **Blank extraction1** | 463 | 186 | 166 | 134 | 69 | 64 |
| **Blank extraction2** | 4364 | 3434 | 3401 | 3364 | 3296 | 3274 |
| **Blank extraction3** | 1245 | 627 | 577 | 514 | 427 | 402 |

**Table 2.** Summary of bacterial Amplicon Sequence Variants (ASV) significantly correlated to higher tetrodotoxin concentrations. Unclass. = unclassified

|  |  |  |  |  |  | **Linear Regression** | |
| --- | --- | --- | --- | --- | --- | --- | --- |
| **Phylum** | **Class** | **Order** | **Family** | **Genus** | **ASVs** | **R^2^** | ***P* value** |
| Acidobacteria | Thermoanaerobaculia | Thermoanaerobaculales | Thermoanaerobaculaceae | Subgroup_10 | ASV3091 | 0.06 | 0.045 |
| Acidobacteria | Blastocatellia  (Subgroup_4) | Blastocatellales | Blastocatellaceae | *Blastocatella* | ASV3444 | 0.21 | <0.0001 |
| Cyanobacteria | Oxyphotobacteria | Unclass. | Unclass. | Unclass. | ASV69 | 0.05 | 0.007 |
| Cyanobacteria | Oxyphotobacteria | Synechococcales | Cyanobiaceae | *Synechococcus_CC9902* | ASV84 | 0.06 | 0.043 |
| Firmicutes | Clostridia | Clostridiales | Peptostreptococcaceae | *Romboutsia* | ASV239 | 0.08 | 0.02 |
| Marinimicrobia  (SAR406_clade) | Unclass. | Unclass. | Unclass. | Unclass. | ASV1911 | 0.23 | <0.0001 |
| Marinimicrobia  (SAR406_clade) | Unclass. | Unclass. | Unclass. | Unclass. | ASV48560 | 0.13 | 0.003 |
| Planctomycetes | Planctomycetacia | Planctomycetales | Gimesiaceae | Unclass. | ASV188 | 0.23 | <0.0001 |
| Proteobacteria | Gammaproteobacteria | Unclass. | Unclass. | Unclass. | ASV12 | 0.16 | 0.001 |
| Tenericutes | Mollicutes | Mycoplasmatales | Mycoplasmataceae | *Mycoplasma* | ASV16 | 0.15 | 0.0021 |
| Tenericutes | Mollicutes | Mycoplasmatales | Mycoplasmataceae | *Mycoplasma* | ASV18 | 0.14 | <0.0001 |
| Tenericutes | Mollicutes | Mycoplasmatales | Mycoplasmataceae | Unclass. | ASV36 | 0.35 | <0.0001 |
| Tenericutes | Mollicutes | Mycoplasmatales | Mycoplasmataceae | *Mycoplasma* | ASV47 | 0.17 | <0.0001 |
| Verrucomicrobia | Verrucomicrobiae | Verrucomicrobiales | Rubritaleaceae | *Roseibacillus* | ASV1308 | 0.13 | 0.003 |
| Verrucomicrobia | Verrucomicrobiae | Verrucomicrobiales | Rubritaleaceae | *Rubritalea* | ASV208 | 0.12 | 0.005 |
| Verrucomicrobia | Verrucomicrobiae | Verrucomicrobiales | Rubritaleaceae | Unclass. | ASV345 | 0.22 | <0.0001 |
| Verrucomicrobia | Verrucomicrobiae | Verrucomicrobiales | Rubritaleaceae | *Roseibacillus* | ASV541 | 0.24 | <0.0001 |

**Table 3.** Summary of bacterial Amplicon Sequence Variants (ASV) corresponding to the core microbiome of *Austrovenus stutchburyi* digestive glands, define as present in at least 70% of all samples. In bold are the genera detected in more than 90% of all samples. Unclass. = unclassified.

| **Phylum** | **Class** | **Order** | **Family** | **Genus** | **Number of ASVs** |
| --- | --- | --- | --- | --- | --- |
| Tenericutes | Mollicutes | Mycoplasmatales | Mycoplasmataceae | ***Mycoplasma*** | 3 |
| Planctomycetes | Planctomycetacia | Gemmatales | Gemmataceae | Unclass. | 2 |
| Fusobacteria | Fusobacteriia | Fusobacteriales | Fusobacteriaceae | ***Psychrilyobacter*** | 1 |
| Spirochaetes | Spirochaetia | Spirochaetales | Spirochaetaceae | Unclass. | 1 |
| Firmicutes | Clostridia | Clostridiales | Peptostreptococcaceae | Unclass. | 1 |
| Firmicutes | Clostridia | Clostridiales | Clostridiaceae_1 | Unclass. | 1 |
| Firmicutes | Clostridia | Clostridiales | Lachnospiraceae | ***Epulopiscium*** | 1 |
| Firmicutes | Clostridia | Clostridiales | Peptostreptococcaceae | *Romboutsia* | 1 |
| Proteobacteria | Gammaproteobacteria | Cellvibrionales | Spongiibacteraceae | Unclass. | 1 |
